# Factors Associated With Low Self-Esteem Among Patients With Hematologic Malignancies: A Cross-Sectional Study

**DOI:** 10.3389/fpsyt.2021.683894

**Published:** 2021-08-19

**Authors:** Junye Yu, Huan Dong, Qi Wu, Ya Yang, Hongying Pi

**Affiliations:** ^1^Chinese PLA General Hospital, Beijing, China; ^2^Aerospace Center Hospital, Beijing, China

**Keywords:** hematology malignancies, cancer, self-esteem, associated factors, coping style, personality

## Abstract

The present study aimed to explore the self-esteem level of patients with hematology malignancies and the associated factors. In this cross-sectional study, we screened patients with hematologic malignancies treated in the Department of Hematology of Aerospace Center Hospital between November 2019 and June 2020. We included 157 eligible patients by convenience sampling. The participants completed questionnaires covering demographic characteristics, loneliness, the coping styles, hope, five personality traits, and self-esteem level. We applied *t*-test, analysis of variance, simple linear regression, and multiple stepwise regression to explore the factors associated with patients' self-esteem. The mean self-esteem score of hematology tumor patients was 26.86 ± 2.34, which was lower than the national norm (*P* < 0.01). The multiple stepwise regression analysis identified maintaining close relationships with others, conscientiousness, extroversion, agreeableness, and positive coping style as factors independently associated with the self-esteem of hematology tumor patients. Patients with hematologic malignancies experience low self-esteem. Factors including maintaining close relationships with others, conscientiousness, extraversion, agreeableness, and active coping style were found to be independently associated with the self-esteem level of these patients. For patients with hematologic malignancies, health providers should apply patient-centered and targeted interventions to improve patients' self-esteem, reduce adverse psychological emotions, and improve their quality of life.

## Introduction

Hematologic malignancies are malignant clonal diseases originating from hematopoietic tissue and have become the fourth most common malignant tumor worldwide (1, 2). This disease is also among the top 10 high-incidence tumors in China, with leukemia being the 9th leading cause of cancer-related deaths after significant increases in its incidence (3, 4).

Due to the progression of the disease and anti-neoplastic treatments, patients with hematological malignancies always experience significant symptoms, including hair loss, vomiting, fatigue, insomnia, dizziness, pain, and even neuropathy (5). Additionally, the stigma of cancer and the despair caused by the prognosis can place great psychological pressure on patients and lead to psychological complications (6). All of the above factors can seriously affect the diagnosis, treatment, and prognosis of the disease, as well as the patient's mood, beliefs, and behavior (7–9).

Self-esteem, as an important sentiment in Chinese native culture (10), is an evaluative and emotional experience of an individual to oneself and represents our sense of self-worth (11). Self-esteem can be threatened by the consequences of various diseases, but high self-esteem is an important prerequisite for successful coping (12). People with high self-esteem have good adaptability (13). However, changes in body image among patients with hematologic malignancies due to the side effects of radiotherapy and chemotherapy can cause low self-esteem (14). Low self-esteem is likely to lead to depression, which can have a negative impact on patients' physical and mental health (13, 15). Studies on self-esteem among patients with hematologic malignancies outside of China have been reported (16–20). A descriptive correlation study reported that Iranian leukemia patients' level of hope was positively correlated with self-esteem (*n* = 85), and appropriate education plans were designed and implemented to help increase hope and self-esteem in leukemia patients (5). However, there is a lack of studies investigating the self-esteem of patients with hematologic tumors in China. Therefore, the present study aimed to explore the self-esteem levels of Chinese patients with hematology malignancies and the associated factors to provide a reference for the development of further targeted measures to improve patients' quality of life.

## Methods

The present study was a cross-sectional study. The Ethics Committee of the Space Center Hospital approved the study (approval #20200108-BSYJS-01). After receiving all study information, each patient signed a consent form before enrollment in this study. This study was reported following the requirements of the STrengthening the Reporting of OBservational studies in Epidemiology (STROBE) statement (21).

### Participants

For this study, we screened patients with various types of hematological malignancies who were hospitalized in the Aerospace Center Hospital between November 2019 and June 2020. The inclusion criteria were: (1) age >16 years, (2) diagnosis with malignant blood disease, (3) stable condition, (4) knowledge of their disease diagnosis, (5) ability to understand and respond to the questionnaire questions, and (6) willingness to participate in this study. The exclusion criteria included: the presence of other severe physical illnesses and mental illness or cognitive impairment.

### Sample Size Calculation

We applied convenience sampling for the questionnaire survey. We calculated the sample size needed for the present cross-sectional study using the following formula:$\text{n }=\;{\left(\frac{Z_{1-\alpha /2}\times \sigma }{\delta }\right)}^{2}$ (22). In this formula, n is the desired sample size; Z_1−α/2_ is a standard value for the corresponding level of confidence; σ is standard deviation; δ is tolerance error. We applied 1.96 to Z_1−α/2_ at a 95% confidence interval (CI). We set a tolerance error (δ) of 0.8, and the standard deviation (σ) was 4.86 (23). The sample size (*n*) required was calculated to be 141. Considering a 10% dropout rate of study participants, 156 hospitalized patients diagnosed with hematologic malignancies were needed.

### Data Collection

After the research assistants obtained the consent of the patients' physicians, a specially trained investigator was responsible for the direction and management of the questionnaire. After the patients or their caregivers signed the informed consent form, the investigator distributed the questionnaire to the patients one-on-one. The investigator explained the purpose, significance, and completion method of the questionnaires to the patients and their families and provided unified instruction to briefly explain the scale. In principle, all questionnaires should have been completed by the patient independently within about 30 min. For patients who could not fill in their answers independently, the investigator could assist them in completing the questionnaires item-by-item without suggestion or guidance. After the completion of the scale, the investigator would check and collect the questionnaires on the spot. If any missing or questionable issues were found, the response would be refilled or verified immediately to ensure the authenticity of the questionnaires.

The basic clinical characteristics of the patients were collected using a pre-tested questionnaire designed by the researchers. The income level in this study was a self-evaluation by patients according to average local income levels supplied in the questionnaire. Family visit refers to the number of family visits during hospitalization. Generally, more family visits reflect more concern by the family about the patient. The other independent variables included the patients' conditions (including the loneliness experienced), coping styles, hope level, and personality traits. The main outcome was the level of patients' self-esteem.

The loneliness experienced was measured using the University of California, Los Angeles Loneliness Scale as revised by Russell et al., which has 20 items, including nine inverse questions (24). The four-point Likert scale (1 = “never,” 2 = “rarely,” 3 = “sometimes,” 4 = “always”) was adopted with a total score of 20–80 points; a higher score reflected a stronger loneliness experience. The Cronbach's coefficient α for the scale was 0.84, and the test-retest reliability was 0.62.

A person's coping style is their typical manner of confronting and dealing with a stressful situation, and this was evaluated among the patients using the Simplified Coping Style Questionnaire (SCSQ) (25, 26). The SCSQ includes a total of 20 items and is mainly used to assess the attitudes and practices of individuals when they encounter difficulties or setbacks. The scale uses a four-point Likert scale method, from “not taken coping” to “often taken coping” in order, corresponding to 0 to 3 points. The Cronbach's α coefficient for the total scale is 0.90, and the Cronbach's α coefficients for the Positive Coping Style Scale and the Negative Coping Style Scale are 0.89 and 0.78, respectively, which indicates good reliability and validity. A higher positive coping score indicates that the subjects are more inclined to adopt positive coping styles, and a higher negative coping score indicates that the respondents are more willing to adopt negative coping styles.

The patient's hope level was measured using the Chinese adapted version of the Herth hope index (HHI) (27). This scale includes 12 items in three dimensions (low hope level, median hope level, and high hope level). The four-point Likert scale method was adopted to assess the condition of participants in terms of a positive attitude toward reality and the future, taking positive actions, and maintaining close relationships with others. A higher score corresponded to a higher hope level. The Cronbach's coefficient α for the scale is 0.78, and the test-retest reliability is 0.86, showing good reliability and validity.

Personality traits refer to people's characteristic patterns of thoughts, feelings, and behaviors, which were evaluated using the Chinese adapted version of the Short Form of the NEO Five-Factor Inventory (NEO-FFI) (28). The NEO-FFI include five dimensions: neuroticism, extraversion, openness to experience, agreeableness, and conscientiousness (29). Each dimension includes 12 items. The five-point Likert scale method was adopted to calculate the total score for each dimension. The internal consistency Cronbach's α coefficient for the total scale is 0.74, and the internal consistency Cronbach's α coefficient for the subscale is between 0.709 and 0.811, indicating the good reliability and validity of the tool.

Self-esteem is an individual's subjective evaluation of their own worth. The level of self-esteem was measured using the Chinese adapted version of the Rosenberg self-esteem scale (RSES) (30, 31). The scale consists of 10 items, including five positive expressions and five negative expressions. The four-point Likert scale method was adopted to measure the overall self-esteem level of the patients. A higher total score on the scale reflected a higher level of self-esteem. The Cronbach's α coefficient for the scale is 0.88, and the test-retest reliability is 0.85, indicating the good reliability and validity of the scale.

### Data Analysis

All data in this study were entered into a computer using Epidata software by two investigators. All statistical analyses were performed using SPSS 23.0 statistical software. The categorical variables were summarized as frequency and proportion, and the continuous variables were summarized by mean ± standard deviation (SD).

We first compared the self-esteem score of the 157 included patients to a national normal model (23). Then, we applied *t*-test or analysis of variance (ANOVA) to check if the basic demographic characteristics of the patients were associated with their self-esteem level. Then, simple linear regression analysis was used to check the associations of the survey findings (e.g., loneliness, hope, coping styles, and personality traits) with self-esteem. Finally, we applied multiple regression analysis to explore the factors independently related to patients' self-esteem. Multiple stepwise regression analysis was performed using a stepwise method to screen independent variables (32). All tests were two-sided, and *P* < 0.05 was considered statistically significant.

## Results

### Basic Characteristics of Participants

The basic characteristics of the included patients were recorded from the completed questionnaires. Of the 157 participants, 56.7% (*n* = 89) were male and 75.2% (*n* = 118) were married. The mean age was 36.45 ± 13.85 years. The majority of the patients had acute non-lymphocytic leukemia (75/157, 47.8%) or acute lymphoblastic leukemia (44/157, 28.3%). Most of the included patients had a high school education, a low economic status, and medical insurance (Table 1).

**Table 1:** Associations between demographic characteristic and self-esteem among the 157 included patients with hematological cancers.

**Variables**	***n* (%)**	**Self-esteem score (mean ± SD)**	^*^P-value
**Age (years)**			
≤ 25	33 (21.0%)	31.39 ± 3.73	0.306
26–49	89 (56.7%)	30.32 ± 3.86	
≥50	35 (22.3%)	31.11 ± 3.63	
**Gender**			
Male	89 (56.7%)	30.57 ± 3.90	0.564
Female	68 (43.3%)	30.92 ± 3.67	
**Marital status**			
Single	38 (24.2%)	30.50 ± 3.87	0.495
Married	118 (75.2%)	30.76 ± 3.87	
Widow	1 (0.6%)	35.00	
**Occupation**			
Worker	13 (8.3%)	30.69 ± 4.92	0.129
Farmer	15 (9.6%)	29.93 ± 3.77	
Clerk	74 (47.1%)	31.45 ± 3.17	
Other	55 (35.0)	29.96 ± 4.16	
**Education level**			
≤ Elementary level	6 (3.8%)	30.00 ± 4.43	0.830
Junior high school level	23 (14.6%)	30.39 ± 3.71	
Senior high school level	40 (25.5%)	30.50 ± 3.78	
≥College or University level	88 (56.1%)	30.97 ± 3.82	
**Family economic status (self-evaluation based on the local income level)**			
Low income	69 (43.9%)	29.48 ± 3.38	<0.001
Moderate income	88 (56.1%)	31.70 ± 3.83	
High income	0 (0%)		
**Residency**			
Rural area	54 (34.4%)	29.28 ± 3.33	<0.001
Urban area	103 (65.6%)	31.49 ± 3.81	
**Duration of illness**			
<1 year	19 (12.1%)	30.95 ± 3.74	0.328
≥1 year but <2 years	105 (66.9%)	29.84 ± 3.71	
≥2 years	33 (21.0%)	31.26 ± 4.18	
**Number of hospitalizations**			
1	21 (13.4%)	30.81 ± 3.53	0.590
2	33 (21.0%)	31.45 ± 3.85	
3	18 (11.5%)	30.89 ± 2.25	
≥4	85 (54.1%)	30.39 ± 4.09	
**Medical expenses payment**			
Paid by patients themselves	28 (17.8%)	29.75 ± 2.93	0.325
Free medical treatment	5 (3.2%)	31.00 ± 2.24	
Medical insurance	124 (79.0%)	30.94 ± 3.99	
**Family visits (during study period)**			
0	22 (14.0%)	31.45 ± 2.91	0.578
1–2	6 (3.8%)	30.00 ± 3.63	
≥3	129 (82.2%)	30.64 ± 3.94	
**Disease subtypes**			
Acute lymphoblastic leukemia	44 (28.3%)	31.35 ± 3.72	0.284
Acute non-lymphocytic leukemia	75 (47.8%)	31.02 ± 3.34	
Chronic leukemia	8 (5.1%)	30.23 ± 3.33	
Others	30 (19.1%)	31.04 ± 3.87	

* *P-value from t-test or ANOVA for associations of the self-esteem scores according to each demographic characteristic*.

### Self-Esteem Level of the Included Patients

Among the 157 included patients with hematologic cancer, the mean self-esteem score was 26.86 ± 2.34, which was lower than the national norm (28.75 ± 4.86; *P* < 0.01) (23). The results of comparisons of the basic demographic characteristics of the 157 patients according to their self-esteem scores are presented in Table 1. The self-esteem level of low-income patients was significantly lower than that of high-income patients (*P* < 0.01). Additionally, rural patients' self-esteem level was significantly lower than that of urban patients (*P* < 0.01).

Simple linear regression analysis was performed to identify factors associated with patients' self-esteem (Table 2). Coping style, hope, five personality traits, and loneliness were found to be associated significantly with patients' self-esteem level (*P* < 0.01).

**Table 2:** Linear regression results for factors associated with patient self-esteem (*n* = 157).

**Independent variables**	**Beta**	**95% confidence interval**	***F***	***P*-value**
**Simple linear regression**				
Loneliness scale	0.500		51.539	<0.001
Hope level	0.551		67.592	<0.001
Positive attitude toward reality and the future	0.430		35.066	<0.001
Active coping styles	0.408		30.900	<0.001
Maintaining close relationships with others	0.617		95.048	<0.001
Positive coping style	0.444		38.094	<0.001
Negative coping style	−0.220		7.889	0.006
Neuroticism	−0.454		40.137	<0.001
Extraversion	0.534		61.845	<0.001
Openness to experience	0.180		5.215	0.024
Agreeableness	0.443		37.867	<0.001
Conscientiousness	0.536		62.546	<0.001
**Multiple linear regression**				
Active emotion-focused coping style	0.491	[0.871, 1.538]	31.862	<0.001
Conscientiousness	0.402	[0.183, 0.367]		<0.001
Extraversion	0.273	[0.114,0.312]		<0.001
Number of hospitalizations	−0.219	[−1.086, −0.373]		<0.001
Medical expenses payment	−0.196	[−1.517, −0.406]		0.001
Agreeableness	−0.239	[−0.363, −0.073]		0.004
Positive coping style	0.166	[0.355, 2.530]		0.010
Duration of illness	−0.113	[−1.179, −0.045]		0.035

*Beta is Standardized Coefficient. Adjusted R Square is 0.613. P < 0.01*.

The multiple linear regression analysis results for factors independently associated with patients' self-esteem are present in Table 2. Maintaining close relationships with others, conscientiousness, extraversion, agreeableness, active coping style, number of hospitalizations, payment method for medical expenses, and length of illness were found to be independently associated with the self-esteem of hematological cancer patients.

## Discussion

In the present cross-sectional study, the mean self-esteem score of patients with hematologic malignancies was 26.86 ± 2.34, which is significantly lower than a previously reported normal score for the national population of China (31). The occurrence of a malignant tumor is a major negative life event (33). The diagnosis of a malignant tumor and the pain of the cancer itself constitute a strong reminder of the possibility of death, easily leading to psychological problems such as depression and death anxiety in patients (34). The low level of the self-esteem among the patients included in the present study may be due to the lack of health education about their disease and misunderstanding of their disease progression and treatment. According to a survey study, blood disease is a risk factor for death anxiety, and good self-esteem can effectively alleviate death anxiety (35). According to the results of Yang et al., a certain amount of psychological counseling based on conventional treatment can enhance the confidence of patients with hematological malignancies that they will be cured, thereby improving their quality of life (36).

In the present study, evaluation of the individual demographic characteristics of the included patients showed that with more or longer hospitalization, self-esteem decreased (*P* < 0.001).

Hope is a multi-dimensional psychological force that can motivate patients to adopt a positive attitude and help them overcome anxiety, fear, and other bad emotions during treatment (37). The present study showed that maintaining close relationships with others was associated with a better level of self-esteem than not maintaining close relationships (*P* < 0.001). Maintaining an intimate relationship with others helps patients obtain sufficient social support, including mental or material help from family, friends, etc., which can buffer stress and maintain a good emotional experience (38). Zhao et al. reported that the social support of patients with hematological disorders is negatively correlated with anxiety and depression. The more social support patients receive, the less negative emotions such as anxiety and depression they will experience, and the higher their level of self-esteem will be (39). Good social support can improve the self-esteem of patients with hematological tumors, reduce their psychological pressure, and promote improvement of their disease (40). Li et al. pointed out that the combinations of cultural and sports activities and personalized health education can effectively improve patients' ability to maintain intimate relationships with others and increase the self-esteem of patients (41). Therefore, in practice, we recommend efforts to achieve good communication between patients with hematologic malignancies and their doctors and to encourage good emotional support from family members to directly improve the level at which the patient maintains close relationships with others, thereby increasing their self-esteem level.

A positive coping style can improve people's mood and improve their ability to deal with issues. On the other hand, a negative coping style makes an individual gradually sink, unwilling to accept facts, which damages the patient's physical and mental health and is not conducive to later recovery and treatment (42, 43). The results of the present study suggest that the active coping style of patients is an important factor associated with the self-esteem level of hematological cancer patients (*P* = 0.01). People with high self-esteem are more inclined to adopt active coping methods such as problem-solving and help, while those with low self-esteem are more inclined to adopt negative coping styles such as self-blame, fantasies, and avoidance (44). Conversely, adopting positive coping styles can enable individuals to get more social support and help, which is conducive to problem-solving, stress relief, and higher life satisfaction (45). Therefore, in practice, when patients are faced with setbacks, encouraging and guiding them to adopt positive coping styles is conducive to the individual's good adaptation to the event, prompting patients to make more positive evaluations of their own condition and experience higher life satisfaction.

Loneliness is a psychological state in which the individuals are dissatisfied with their interpersonal relationship status, and it is often accompanied by negative emotional reactions such as helplessness and depression (46). The unbearable sense of mental loss hurts cognition and behavior and is an important risk factor for mental and physical health problems (6). Loneliness has been shown in several studies to be negatively associated with recovery from a number of chronic diseases, particularly cancer. Additionally, loneliness increases the risk of physical and mental health problems and increases premature mortality and national mortality. It was reported that loneliness affects patients' self-esteem through feelings such as helplessness and depression (47, 48). In the present study, loneliness was negatively associated with patients' self-esteem on simple linear regression analysis, but not on the multiple linear regression analysis. The reason is unknown, and thus, further investigation and analysis on this topic is warranted.

The five personality traits are human beings' relative stability traits, which maintain a relatively stable worrying tendency or worrying habits with individual differences at different times and in different situations (49). Conversely, when facing the same pressure, individuals with low self-esteem attribute the event to self-ability limitations, and individuals with high self-esteem attribute the event to external causes (50, 51). The present study shows that conscientiousness, extraversion, and agreeableness are factors associated with the self-esteem of hematological cancer patients. Personality traits are relatively stable over time and have cross-contextual stability. However, such characteristics vary from person to person, which is responsible for the differences in personality between people. Self-esteem refers to the evaluation made or usually held by individuals. It is a spiritual need, the core of personality, and the core of mental health. Therefore, each person's personality is different and stable, which means that the level of self-esteem also varies from person to person and cannot be adjusted (52).

To our best knowledge, this is the first study to explore the factors associated with the self-esteem of patients with hematologic malignancies. There are several limitations for the present study. First, as the present study is a cross-sectional study, not a cohort study, we cannot conclude that the factors we found associated with patients' self-esteem are independent influencers. Next, in the present study, we included patients both at the beginning of their diagnosis stages and who were receiving treatment with chemotherapy/radiotherapy to capture any possible mental features in this unique group. A subgroup analysis of patients in different treatment stages will be performed soon. Finally, the included patients came from a tertiary hospital in Beijing, which resulted in the patients having high education levels. Because Beijing is the capital of China, there are many people with high education qualifications. Additionally, Beijing attracts many people with high education qualifications seeking medical treatment. The scope of the study will be expanded in later research efforts, and the results of the study will be optimized. Further research with a cohort design or even a randomized controlled design is needed to confirm our findings.

## Conclusion

Patients with hematologic malignancies experience low self-esteem. Factors, including maintaining close relationship with others, conscientiousness, extraversion, agreeableness, and active coping style, were found to be independently associated with the self-esteem level of patients with hematologic malignancies. For patients with hematologic malignancies, health providers should apply patient-centered and targeted interventions to improve patients' self-esteem, reduce adverse psychological emotions, promote overall well-being, and improve their quality of life.

## Data Availability Statement

The raw data supporting the conclusions of this article will be made available by the authors, without undue reservation.

## Ethics Statement

The studies involving human participants were reviewed and approved by The Ethics Committee of the Space Center Hospital approved the study (approval # 20200108-BSYJS-01). The patients/participants provided their written informed consent to participate in this study.

## Author Contributions

JY designed the study and revised the manuscript. HD and QW conducted all the data analyses and wrote the manuscript. YY collected the data. HP provided guidance and assistance in the topic selection design and implementation, and thesis writing process. All authors read and approved the final manuscript.

## Conflict of Interest

The authors declare that the research was conducted in the absence of any commercial or financial relationships that could be construed as a potential conflict of interest.

## Publisher's Note

All claims expressed in this article are solely those of the authors and do not necessarily represent those of their affiliated organizations, or those of the publisher, the editors and the reviewers. Any product that may be evaluated in this article, or claim that may be made by its manufacturer, is not guaranteed or endorsed by the publisher.
